# Shape Memory Alloy Capsule Micropump for Drug Delivery Applications

**DOI:** 10.3390/mi12050520

**Published:** 2021-05-06

**Authors:** Youssef Kotb, Islam Elgamal, Mohamed Serry

**Affiliations:** Department of Mechanical Engineering, American University in Cairo, New Cairo 11835, Egypt; youssefmohamed@aucegypt.edu (Y.K.); islamelgamal@aucegypt.edu (I.E.)

**Keywords:** shape memory alloy, micropump, drug delivery, microfluidics, bicuspid valve, capsule pump, high throughput

## Abstract

We introduce a shape memory alloy (SMA) actuated micropump optimized for drug delivery applications. The proposed novel design integrates a built-in replaceable drug reservoir within the pump package forming a self-contained preloaded capsule pump with an overall pump volume of 424.7 μL. The new design results in a compact, simple, and inexpensive micropump and reduces the probability of contamination with attained almost zero dead volume values. The pump consists of NiTi-alloy SMA wires coiled on a flexible polymeric enclosure and actuated by joule heating. Unlike diaphragm and peristaltic SMA micropump designs that actuate transversely, our design is actuated longitudinally along the direction of the highest mechanical compliance resulting in large strokes in the order of 5.6 mm at 27% deflection ratio, actuation speed up to 11 mm/s, and static head pressures up to 14 kPa (105 mmHg) at 7.1 W input power; thus, high throughputs exceeding 2524 μL/min under free convention conditions could be achieved. A model was developed to optimize the pump’s geometrical parameters and the enclosure material. The model concluded that low stiffness enclosure material combined with thinner SMA wire diameter would result in the maximum deflection at the lowest power rating. To prove its viability for drug delivery applications, the pump was operated at a constant discharge volume at a relatively constant static head pressure. Furthermore, a design of bicuspid-inspired polymeric check-valves is presented and integrated onto the pump to regulate the flow. Since the built-in reservoir is replaceable, the pump capsule can be reused multiple times and for multiple drug types.

## 1. Introduction

Implantable drug delivery systems are gaining much attention in recent years due to their advantages over conventional drug delivery methods (e.g., oral and intravenous). They allow the use of higher localized concentrations, thus making the treatment more effective by delivering the drug directly to the tissue. Lessening the spread through the unintended body tissues, and controlling drug release can be optimized for maximum efficiency and a minimum amount of drug used, thus decreasing the cost of treatment. This also enables drug delivery to immunologically isolated parts of the body (e.g., cornea), unreachable with conventional drug delivery [1].

Although controlled passive-release drug delivery devices such as microencapsulation [2], bio-adhesives [3], polymer implants [4], and transdermal patches [5] are currently the most dominant drug delivery methods; they are designed to deliver small amounts of the drug gradually and at a constant rate through difference in concentration between the drug and the surrounding tissue, but not capable of delivering the drug in a nonlinear fashion, i.e., they are nonprogrammable and cannot deliver the drug on-demand. Moreover, some clinical situations (e.g., delivery of hormones, anticancer agents, and vaccines) necessitate either external control and configurability of the drug delivery rate or dose beyond the existing controlled passive-release drug delivery capabilities methods [6,7].

Thus, there has been an increased interest in miniature active-controlled drug delivery systems utilizing wearable or implantable micropumps capable of delivering microliters of medicine on demand by forcing the delivery through pressure difference rather than the concentration difference between drug and the surrounding tissue.

Smart material actuators have gained enormous appeal for manufacturing wearable and implantable micropumps due to their high energy density [8,9]. However, smart material actuators generally have several shortcomings involving either stroke or pressure limitations [10].

Shape memory alloy (SMA) actuators are based on NiTi metallic alloys that can recover permanent strain by changes either in stress, temperature, or a combination of both. SMA generates large strain when temperature changes due to diffusionless transformation (twinning/detwinning) from martensite to austenite phases [11,12].

There are several advantages to SMA-actuated micropumps, including (i) being a direct drive actuator with solid-to-solid phase transformation (i.e., absence of friction, no moving or reductions mechanisms required, and noiseless), which also leads to the device being compact and more reliable and more suitable for miniaturized systems, (ii) large strokes can be realized, (iii) operating quasi-statically and independent of patient motion or body orientation, (iv) can provide high power-density; thus high flow rates and high pressures can be attained with miniaturized pumps, (v) excellent corrosion resistance and biocompatibility [10,13].

On the other hand, the main disadvantage with SMA micropumps is their low actuation frequency since the phase change in SMA is achieved by heat exchange with the surroundings heat source or a heat sink. Thus, the heating and cooling rates determine the frequency response of the SMA pump [14].

Several thin-film SMA strip and diaphragm type micropumps with high reciprocation rates have been widely reported in literature [13,15]. One thin-film SMA diaphragm pump is capable of pumping up to 50 μL/min [16,17]. A reciprocating SMA peristaltic pump designs which can pump up to 1000 μL/min of fluid were also reported [18,19,20]. However, both the diaphragm and peristaltic designs commonly used in SMA micropumps do not allow large displacements, thus limiting the pump’s discharge volume, throughput, and static head pressure.

One application of SMA micropump is for chemical delivery to biochemical integrated circuit chips [21,22]. However, the use of SMA micropumps for implantable drug delivery applications necessitates online configurability, operating with no vibrations and at a low energy budget (i.e., battery-powered) and being small in size.

Therefore, in this paper, we present a new design of a high-power density resistively actuated SMA capsule micropump optimized for drug delivery applications. Unlike the typical diaphragm and peristaltic designs, our pump is actuated linearly in the most compliant direction allowing large displacements, large discharge volume, and high throughput. The pump’s capsule design integrates a replaceable drug reservoir that can be preloaded with the desired drug within the pump package, increasing the fill-factor and reducing the overall footprint (i.e., the overall area occupied by the pump, including the actuation mechanisms, the valves, and the drug reservoir. Our pump integrates all these components in one self-contained package, thus reducing the overall occupied area). The pump structure is made out of an elastic rubber material that acts as a spring and is useful in recovering from the contraction after the actuation (i.e., from the detwinned to the twinned phase). The structure is also flexible, insulated, and biocompatible for drug delivery. The reservoir is replaceable, which means that the pump can be reusable many times and for different drug types. This makes the whole package compact, efficient, cheap, and compatible for drug delivery applications.

## 2. Principle of Operation

### 2.1. Pump Chararacteristics

The pump concept is based on winding SMA wires on a flexible polymer capsule. Upon heating the spring, SMA converts the heat energy into a linear displacement that leads to injecting the drug through a one-way valve to the desired tissue. Upon running off the current, the detwinning is obtained by the polymer capsule’s stiffness, thus resetting the pump to the initial position. The NiTi SMA wires with a round cross-section measuring 0.25, 0.4, and 0.5 mm in diameter were coiled on 6-mm diameter polymeric enclosure (i.e., capsule) spring diameter (i.e., 21.2 mm^2^ cross-sectional area, 424.7 mm^3^ volume) were selected to increase the heat transfer rate compared to the polymer enclosure. The actuation frequency of the SMA spring is dependent on the pulse of the voltage and the current from the supply. Each pulse’s on-time results in the pump’s contraction, whereas during off-time, the pump recovers its position and takes up the drug from the integrated reservoir.

### 2.2. Pump Operation

**Figure 1:**
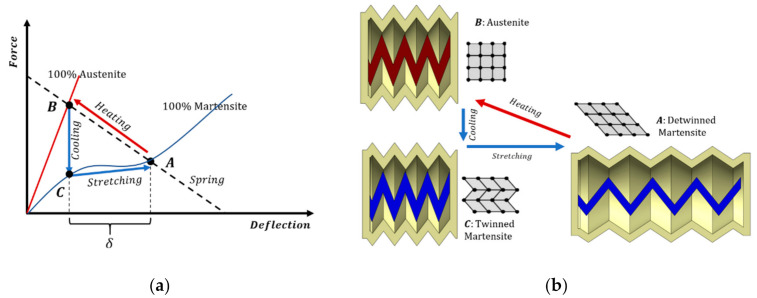
(**a**) Force-deflection curves for the shape memory alloy (SMA) wire in its 100% austenite and 100% martensite phase, and the silicone enclosure in relation to them; (**b**) SMA’s crystalline arrangement during the different operation stages.

To use the SMA coil as an actuator, another element needs to be added to apply a restoring force. Therefore, the spring can be compressed through resistive heating. However, to stretch the spring back to its stretched form, an opposing spring must be added to apply a restoring force. In this design, the spring is the silicone enclosure. At any point in the operation, the pump’s instantaneous deflection is the point at which the coil and silicone enclosure apply the same force, as illustrated in Figure 1. Since the SMA coil’s force-deflection behavior changes with the instantaneous SMA material phase, the intersection points to move, causing a deflection in the pump.

When the current passes through the SMA wire, its temperature will rise by the joule heating effect. This causes a phase change in the SMA spring to the austinite phase. The force-deflection curve of the SMA spring in the austenite phase is significantly larger than that of the martensite. This causes the spring to contract along with the silicone enclosure. This deflection causes fluid to be pumped out equal to the change in volume. After this contraction, the current is turned off and left to cool down. As it cools down, SMA will attempt to restore back to the twinned martensite phase. This phase has a lower force-deflection curve, so the stiffness of the deformed silicone enclosure will recover and stretch the coil back to its initial position. As the coil is stretching, it gradually changes the inner structure to detwinned martensite, as shown in Figure 1b. During this phase, fluid is pulled into the pump from the built-in drug reservoir and ready to pump the drug in the next cycle. The maximum fluid pressure pumped during compression is caused by the difference in force between the SMA coil in its austenite phase and the silicone container. During the expansion phase, the pump’s negative pressure pulls fluid in from the reservoir with force applied by the silicone container.

### 2.3. Valve Design

**Figure 2:**
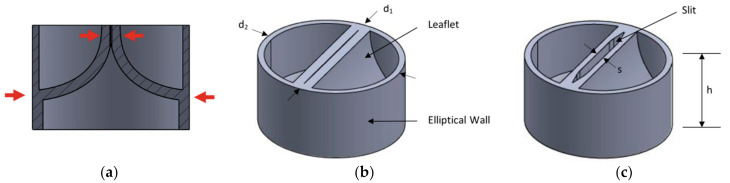
3D-printed valve (**a**) cross-section, (**b**) valve in the closed-mode, and (**c**) valve in the open-mode, where h = 1.5 and 3.5 mm, s = 0.2–0.5 mm, d_1_ = 3.5 and 6 mm, and d_2_ = 4 and 7 mm for the inner and outer valves, respectively.

The valve’s design is inspired by the bicuspid aortic valve. It is composed of two flexible leaflets opposing each other connected to a flexible cylindrical wall (Figure 2). To ensure that the valve is fully closed in the absence of fluid flow, the leaflets are spatially constrained to push against each other. The valve’s elliptical shape makes it preloaded such that it is compressed in the direction normal to the leaflets and constraining any leakage. The leaflets are curved in such a way to allow internal pressure to push the leaflets apart, and external fluid pressure pushes the two leaflets together during the pumping cycle. The valve is printed as a single part by stereolithography 3D printing with desired stiffness using Formlab’s Shore 80 A flexible resin.

## 3. Modeling and Parametric Study of the SMA Pump

Shape memory alloys behave in a complicated nonlinear thermomechanical system when subjected to temperature and load changes. There have been many theoretical and empirical models developed for simulating this behavior [23]. However, accurately computing these equations requires complex numerical methods and a thorough understanding of the environment surrounding the material. For this device, we are mainly interested in the maximum deflection attainable by the pump with specified SMA wire and coiling parameters (i.e., wire diameter and number of coil turns) and the polymeric enclosure parameters.

Since we are interested only in the pump’s minimum and maximum deflections, there is no need to compute the intermediate deflection values. Thus, only the force-deflection curve of the SMA spring in both its austenite and martensite phases needs to be computed to know these values. To do so, we assumed that the material is composed of 100% austenite when heated and 100% martensite when left to cool. Moreover, since the pump is stationary at these two points, the fluid effects on the pumping forces can be neglected.

The SMA material’s behavior in its austenite phase is similar to a typical metal’s behavior; with a constant stiffness in its elastic region up until its maximum strain, then it goes into plastic deformation. Its behavior in its martensite phase, however, is much more complex. When sufficient stress is applied, the material’s inner lattice structure changes from twinned to detwinned, causing much larger deformation. This transformation happens gradually as the stress increases between two critical values ($\tau_{Ms}^{cr},\tau_{Mf}^{cr}$), causing a change in the percentage of detwinned martensite ($\xi_{St}$) from zero to 100%. The material behaves elastically below and above these critical points until it goes into the plastic deformation region. The SMA material itself has many properties that determine its thermomechanical behavior, defined in Table 1.

The relationship between strain and detwinned martensite percentage was empirically defined in Brinson’s thermomechanical model of shape memory alloys, as described in Equation (1) [24].

(1) $$\xi_{St}=0.5\cos\left(\frac{\pi \left(\tau -\tau_{f}^{cr}\right)}{\tau_{s}^{cr}-\tau_{f}^{cr}}\right)+0.5$$

Shaping the SMA material in the form of spring allows it to deform to much greater lengths. The spring itself can be fully defined through four design parameters: the number of active turns (*n*), initial pitch angle (*α*), wire diameter (*d*), and coil diameter (*D*), or spring index (*C* = *D*/*d*). A model was developed to find the force-displacement relationship of an SMA spring actuator [25]. This model takes into consideration the torsional force across the wire and the change of the outer diameter as the spring deflects. Through this model, the SMA spring’s reaction force for various deflections is computed in both its martensite and austenite phases [25]. The force of the SMA spring in its martensite phase can be found in Equation (2).

(2) $$F_{M}=\frac{G_{M}d^{4}}{8D^{3}n}*\left(\frac{\cos^{3}\alpha_{i}\;}{\cos^{2}\alpha_{f}+\frac{\sin^{2}\alpha_{f}}{1+\nu }}\right)*\delta -\frac{{\pi d}^{3}}{8D}G_{M}\gamma_{L}\xi_{St}$$

Finally, to find the deflections in the cycle, the intersection points between the spring and the silicone container’s force-displacement curves are found. The silicone’s stretching force was found experimentally by measuring both displacement and force as the part is compressed. The curve was found to be highly linear in the operating range, so it was assumed to have constant stiffness. Since the displacement of the silicone is equal in magnitude and opposite in direction, the relationship can be defined as follows:

(3) $$\delta_{silicone}=d_{i}-\delta_{spring}$$

**Figure 3:**
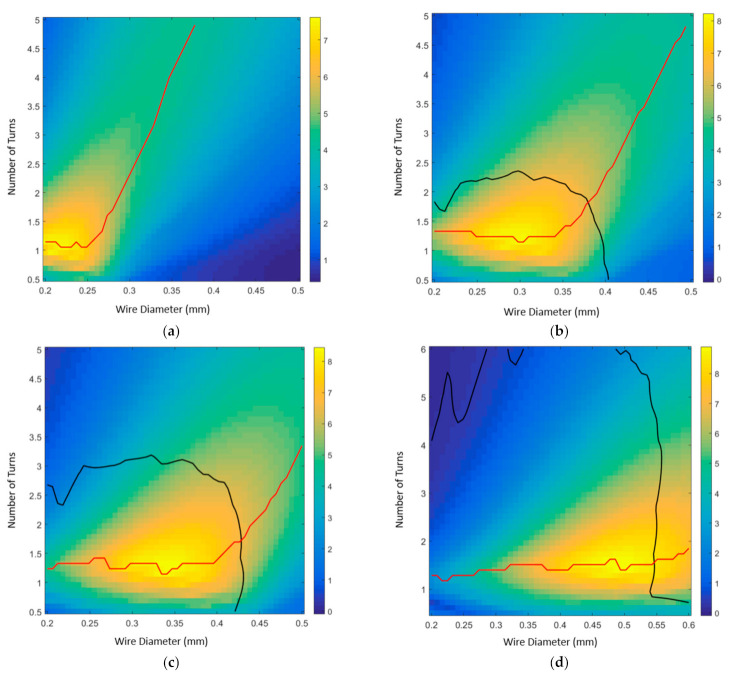
The maximum deflection (in mm) as a function of the wire diameter (x-axis, in mm) and the number of turns (y-axis). The regions inside the black boundary represent the designs that cause the SMA coil to deform plastically, whereas the regions outside the black boundary represent the elastic deflections. The red trend lines follow the peak points at which there is maximum displacement for the wire thickness values. Enclosure material curves for (**a**) Ecoflex 00-10 (**b**) Ecoflex 00-30 (**c**) Ecoflex 00-50 (**d**) PDMS.

This system of equations was solved on MATLAB code to find the force values for an array of deflection values for the spring in its martensite and austenite phases and the enclosure material. The code finds the intersection of these curves to find the maximum deflection values. The material properties of the SMA material were obtained by experimentally measuring the force-displacement behavior of the SMA coil (values given in Table 1). The maximum deflection computed through this model was found to be sufficiently close to the experimental results. This model proved to be useful in predicting nonviable designs which would cause the spring to deform plastically. The model was then used to optimize the design values of the SMA coil, namely the number of active turns and wire diameter. The spring diameter is set to the pump diameter, and its initial pitch angle is approximately zero as it is tightly wound. The model results are summarized in Figure 3 showing the effect of wire diameter, number of turns, and enclosure material on the pump deflection. The region within the black curve represents the designs that would fail plastically (i.e., undesirable), whereas the regions outside would favorably fall in the elastic range. The red trend lines show that as we increase the number of turns, the optimum design would require a larger wire diameter, which is not desirable due to the increased current and power consumption. The curves also show that as we increased the enclosure material stiffness (i.e., ranging from Ecoflex 00-10 to Polydimethylsiloxane (PDMS)), the peak deflection (i.e., the hotspot on the curves) occurred at larger wire diameters. Accordingly, it can be concluded from this model that larger material stiffness should be avoided, whereas lower stiffnesses such as Ecoflex 00-10 would be the best choice. However, using Ecoflex 00-10 would result in several practical issues related to the structure’s mechanical robustness and the number of pumping cycles. Therefore, the optimal enclosure material choice under these conditions was found to be Ecoflex 00-30.

## 4. Materials and Methods

### 4.1. Materials

NiTi wires of diameters 0.25, 0.4, and 0.5 mm with the transition temperature of 45 °C were obtained from Kellogg’s Research Lab, USA. As discussed earlier, designing an SMA micropump requires a flexible structural material enclosure material with the correct stiffness to restore the SMA phase. Moreover, the enclosure material must be easily molded and cured at a temperature lower than the austenite transition temperature. Also, based on the above-mentioned model results, it has to have a small young’s modulus to minimize the SMA wire size and power consumption. Therefore, Ecoflex 00-30 platinum silicone was chosen for its convenient mechanical properties, namely its large elongation, ease of molding due to low mixed viscosity, short room temperature curing time (c.f. Table 2) and biocompatibility [26,27]. It was chosen over Ecoflex 00-10 grade due to its strength and mechanical robustness, which will allow the pump to work over larger number of cycles. Functionally, Ecoflex 00-10 is ~4.7 times more viscous than 00-30 (rated at 14 Pa·s) [28], which compromises the molding of the fine pump features. Moreover, Ecoflex 00-10 has 40% less tensile strength than 00-30 (rated at 0.83 MPa) and 42% less tear strength (3.85 N/mm versus 6.65 N/mm). This will affect the device’s reliability since the pump’s operation requires stretching (i.e., elongation >27%) the polymer many times and rebounding to the original position, which increases the possibility of tearing at the 00-10 lower tear and tensile strengths.

Ecoflex 00-30 was also chosen over PDMS based on the model results to allow the usage of a thinner wire diameter and reduce the current and power consumption.

### 4.2. Pump Fabrication

**Figure 4:**
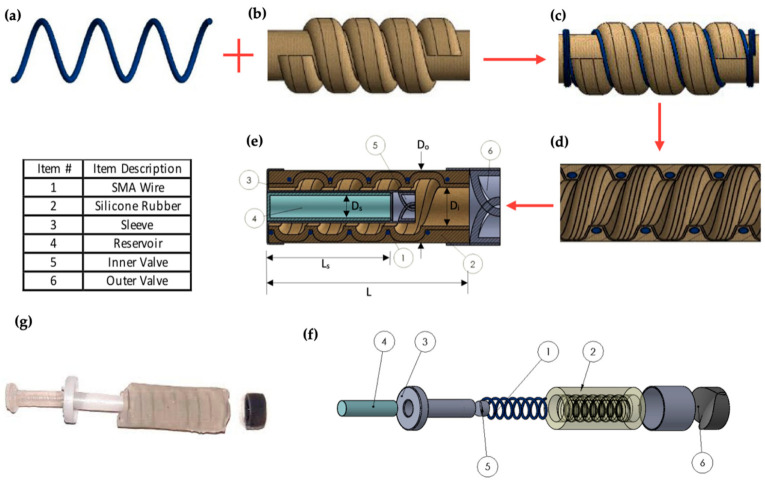
(**a**–**e**) Process flow diagram, (**f**) exploded view of the pump, and (**g**) image of the fabricated pump showing the different components, where L = 20 mm, L_s_ = 14.6 mm, D_o_ = 9.4 mm, D_i_ = 5.2 mm, D_s_ = 3 mm.

A four-step process was optimized to fabricate the SMA micropumps, as shown in Figure 4. As shown in the figure, Ecoflex was shaped in a 3D printed mold to form the inner pump structure. In the next step, the SMA wires were coiled along the inner structure’s helical grooves (c.f. Figure 4c) with the desired number of turns (3, 4, 6, and 8 turns). Next, the outer Ecoflex capsule is shaped in a 3D printed mold and assembled with the inner structure as shown in Figure 4d. The drug reservoir is shaped using Ecoflex 00-30 in one step in an aluminum mold. The inner and the outer valves are each printed as a single part through Stereolithography with flexible 80 A resin by Formlabs, USA. A sleeve (part#3) was used for containing the replaceable reservoir and the inner valve that connect the reservoir to the main pump chamber. It also functions as a fixed support to the pump as it fixes one end of the pump for the SMA spring to contract, thus pushing out the pump’s volume without squeezing the reservoir. Finally, the pump structure, the sleeve, the reservoir, and the valves are assembled, as shown in Figure 4e–g show schematics of the pump assembly and actual picture of the developed pump components, respectively.

### 4.3. Characterization

The pump’s displacement, discharge, volume flow rate, static pressure head, and the Ecoflex’s and SMA’s force-displacement responses were experimentally characterized using force (resolution = 0.03 N) and linear motion (resolution = 0.0078 mm) sensors by PASCO, USA.

## 5. Results and Discussion

The results of the experimental characterization of the pump are illustrated in the following sections.

### 5.1. Maximum Displaced Volume

**Figure 5:**
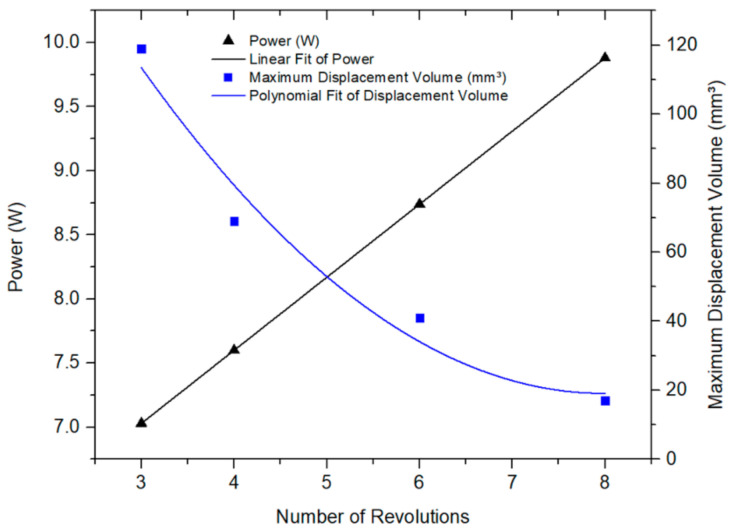
Pump discharge and input power vs. number of SMA wire turns.

**Figure 6:**
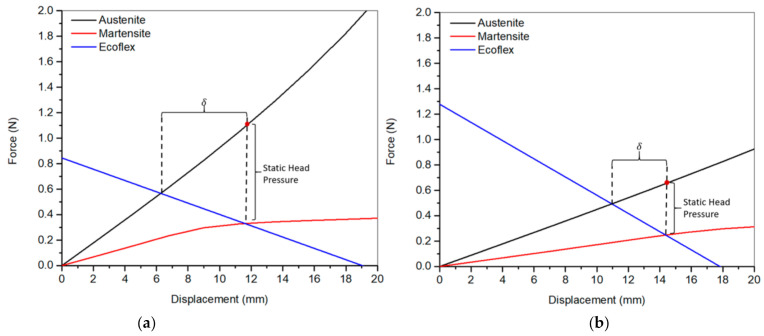
Force-displacement curve of the SMA wire in austenite and martensite phases, and the Ecoflex enclosure for (**a**) 3-turn and (**b**) 6-turn pumps, at wire diameter = 0.4 mm. The pump’s full range of motion from martensite to austenite is represented by δ.

Figure 5 illustrates the effect of the number of turns on the maximum discharge volume at constant current (1.9 A) and SMA wire diameter = 0.4 mm and different input powers. As shown in the graph, it was observed that increasing the number of turns decreased the maximum displaced volume while the input power increased (i.e., the voltage increases). This can be explained further by examining Figure 6, which depicts the force-displacement curves of the SMA wires in both phases, austenite and martensite, and its intersection with the Ecoflex (silicone) force-displacement curve as estimated from the model. As shown in this figure, the intercept points represent the full phase motion between martensite and austenite. The stiffness of the silicone dropped while the SMA wire’s stiffness increased due to a decrease in the overall length allowing for a broader range of motion between martensite and austenite indicated as δ in Figure 6. The opposite happens in the case of 6 turns, resulting in a shorter full motion range.

It has also been observed that decreasing the voltage and current gradually increases the time taken to heat the SMA springs, reaching up to a point where the energy applied to the SMA spring to heat up equals the energy lost to the surroundings by heat convection. Therefore, the time used by the pump to actuate increases, and also the maximum deflection and displaced volume decrease for each pump.

### 5.2. Effect of Drive Frquency on Flow Rate

**Figure 7:**
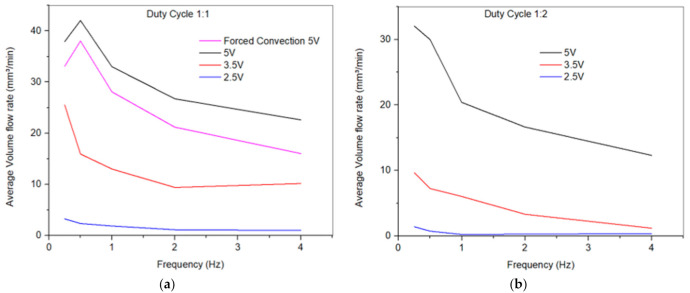
Volume flow rate vs. frequency at 2.5, 3.5, and 5 V for the 3-turn pump at (**a**) duty cycle 1:1 (**b**) duty Cycle 1:2. Figure (**a**) also shows forced convection cooling mode at 5 V.

**Figure 8:**
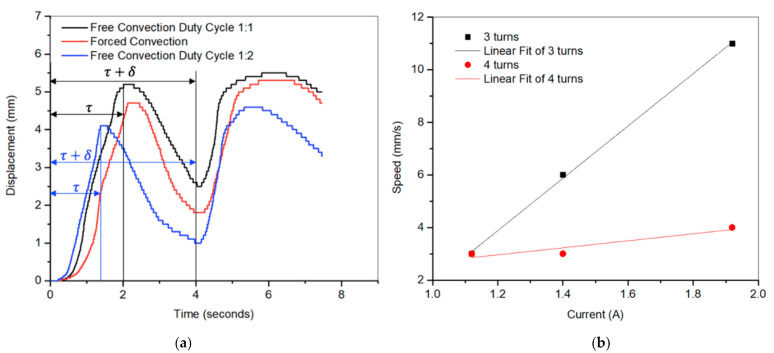
(**a**) Pump displacement vs. time at duty cycles of 1:1 and 1:2 and forced convection conditions. (**b**) Speed vs. input current for the 3- and 4-turn pumps. Cycle time = 4 s, for the 1:1 cycle, τ=2 s, and δ=2 s. For the 1:2 cycle, τ=1.33 s, and δ=2.66 s.

Figure 7a,b shows the flow rate versus frequency for the 3-turn pumps with duty cycles 1:1 and 1:2, respectively. It was observed that increasing the duty cycle decreased the maximum flow rate and the pump discharge volume. Similarly, it was observed that cooling the pump using forced convection (convection coefficient, h ≈ 79 W/m^2^. K) did not significantly affect the device. These two phenomena can be explained further through Figure 8a. As we increased the duty cycle, the device was energized 33 percent of the time, which resulted in extended cooling time and deeper trough. This is a result of the continuous rapid heat dissipation of the actuator. The SMA spring actuator was not heating up enough to give the large stroke required for larger volume flow rate. Therefore, for the same amount of power, the displacement did not rise to a comparable value as in the duty cycle of 1:1, whereas operating in the forced convection mode would reduce the cooling time; on the other hand, as demonstrated in the red curve in Figure 8a, this would also result in reducing the heating efficiency and the overall pump’s contraction (i.e., stroke), resulting in a lower flow rate.

Further, it was observed under the duty cycle 1:1 condition that increasing the frequency beyond 2 Hz did not substantially impact the maximum flow rate (i.e., the curve flattened out because of the short rise time and the shortage of time for the actuator to cool down.). Whereas, below 2 Hz, there will always be an optimum frequency at which the SMA heats up to reach 100% austenite phase and cools down to reach the 100% martensite phase, and the cycle repeats itself. Figure 8b shows that pumping speeds up to 11 mm/s could be attained for the 3-turn devices.

### 5.3. Effect of Wire Diameter and Number of Turns on the Flow Rate

**Figure 9:**
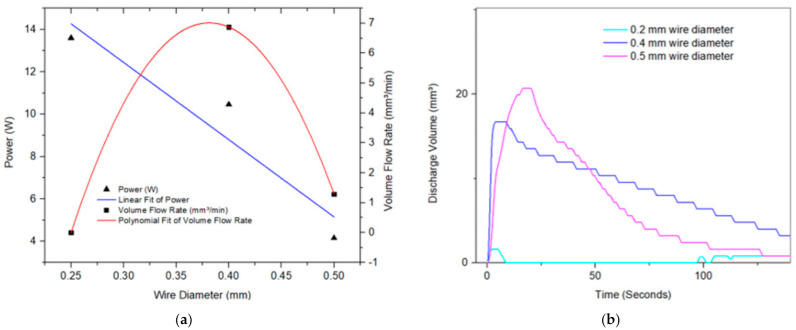
(**a**) Pump’s volume flow rate and input power vs. SMA wire diameter. (**b**) Pump discharge volume at different SMA wire diameters.

**Figure 10:**
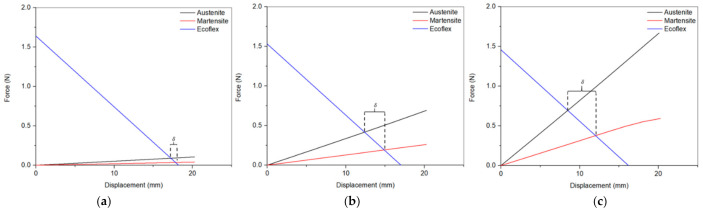
Force-displacement curve of the SMA in austenite and martensite phases, and the Ecoflex enclosure for SMA wire diameters of (**a**) 0.25, (**b**) 0.4, and (**c**) 0.5 mm for the 8-turn devices. The pump’s full range of motion from martensite to austenite is represented by δ.

Figure 9a shows the effect of the SMA wire diameter (i.e., 0.25, 0.4 and 0.5 mm) on the discharged volume over time at constant current (1.9 A). It is observed that while the 0.5 mm wire had the maximum displaced volume (i.e., maximum deflection), the maximum flow rate was achieved at 0.4 mm wire diameter, as shown in Figure 9b. This effect can be explained by examining Figure 10, which shows that the full range of the transformation between the austenite and martensite is the largest at 0.5 mm wire diameter. However, due to the constant current, the input power drops/decreases as we increase the wire diameter (i.e., resistance and voltage decrease), which results in a slower rise and an overall flow rate.

### 5.4. Effect of Number of Turns on Static Pressure Head

**Figure 11:**
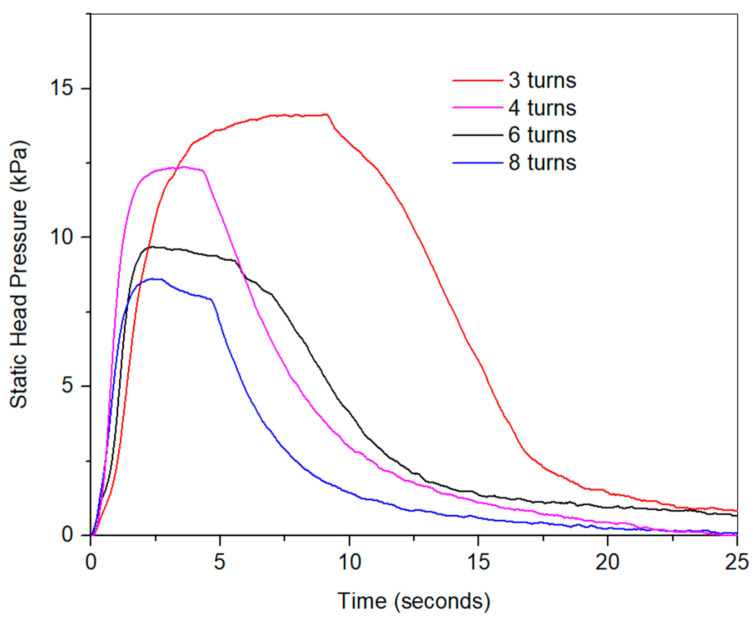
Pump’s static head pressure for the 3, 4, 6, and 8-turn pumps.

Figure 11 illustrates the effect of the number of turns on the pump’s static head pressure. As shown in the figure, the static head pressure increased as we decreased the number of turns. This can also be explained in light of Figure 6, which indicates that the contraction force at the 100% austenite increased as we decreased the number of turns due to the increase in the wire stiffness while decreasing the enclosure stiffness.

### 5.5. Pump Operation

To control the discharge volume, the pump was operated in a limit-switch mode whereby the discharge volume per stroke was kept constant by turning the current on at a constant value until the target discharge volume was reached. Afterwards, the current switched off, allowing the SMA wire to relax to full martensite transformation, and the current was allowed to turn on again.

**Figure 12:**
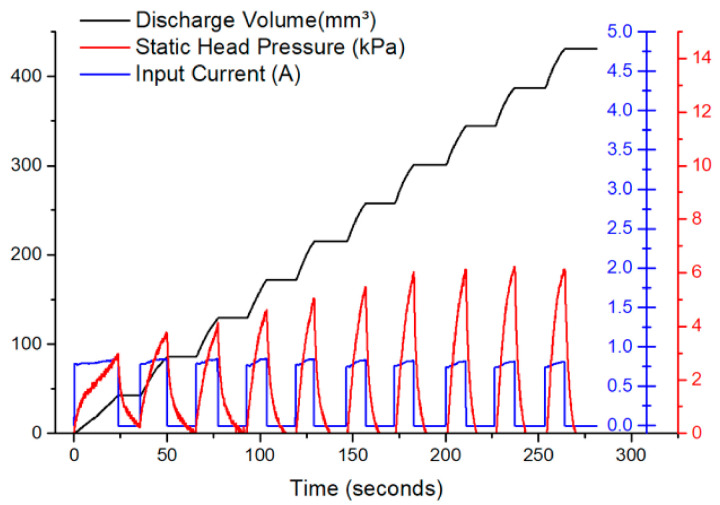
Discharge volume, the static head pressure, and the current profile for the 3-turn pump operating at 790 mA input current in a controlled pump operation mode.

Figure 12 shows the discharge volume, the static head pressure, and the current profile for the 3-turn pump. The target pump discharge was fixed at 43 mm^3^, and the maximum input current was set at 790 mA. It was noted that the static head pressure response was also constant at 6.5 kPa (~48 mmHg) for each stroke, which proves the viability of this pump for drug delivery applications.

Finally, Table 3 compares the proposed pump’s performance at 7.1, 4.2, and 2.9 W against state-of-the-art SMA actuated micropumps. The table demonstrates that at such a small footprint, our pump demonstrates a large stroke, the largest flow rate per watt at 364.52 μL/(watt·min) at 4.2 W, and the largest flow rate of 2.524 mL/min at 7.1 W.

## 6. Conclusions

This paper introduced the design, fabrication, modeling, optimization, and experimental characterization of a novel SMA actuated capsule micropump. The pump’s linear actuation leads to large deflection, high discharge volume, and high static head pressure. A model was developed to investigate the effect of the number of turns and the enclosure material’s stiffness on the pump’s deflection. It was found that enclosure materials with lower stiffness would maximize deflection at the lowest power consumption. The new pump design is optimized for drug delivery applications by introducing a replaceable capsule reservoir and controlling the drug’s delivery at a constant dose and a constant static head pressure. In summary, the device has a wide range of flow rates (from less than 2 to more than 2500 μL/min) that would be suitable for a broad range of drug delivery applications. Also, it was demonstrated that a wide range of pressures up to 14 kPa (105 mmHg) is possible, which well exceeds the back pressure of most of the superficial veins typically utilized for intravenous drug delivery, without compromising the suitability for transdermal drug delivery.

## 7. Patents

The work reported in this paper is the subject of a United States Patent Application no. 63/167783.

## Figures and Tables

**Table 1:** Thermomechanical behavior parameter used in the model.

Parameter	Definition	Value *
$\xi_{St}$	Fraction of detwinned martensite	Varies from 0–1
$\tau_{Ms}^{cr},\tau_{Mf}^{cr}$	Critical starting and ending shear stresses	105 and 215 MPa
$\gamma_{L}$	Maximum residual strain	0.056
$G_{M},\;G_{A}$	Shear modulus of martensite and austenite phases	5.65 and 18.3 GPa

* Experimentally measured values.

**Table 2:** Ecoflex 00-30 material properties (compiled from [28]).

Material Property	Value
Mixed Viscosity [Pa·s]	3
Specific Gravity [g/cm^3^]	1.07
Shore Hardness [ASTM D-2240]	00–30
Tear Strength [N/mm]	6.65
Tensile Strength [MPa]	1.38
Elongation at break [%]	900
Shrinkage [mm/mm]	<0.001
Curing Time at Room Temperature [h]	4

**Table 3:** Comparison table showing our developed pump’s performance relative to state-of-the-art SMA-actuated micropumps.

Reported Work	Pump Volume (mm^3^)	Power (W)	Max Stroke (mm)	SHP * kPa	Transition Temp (°C)	Max Flow Rate (μL/min)	Max Flow Rate Per Watt (μL/(watt·min))	Max Flow Rate Per Pump Volume (μL/(min·mm^3^))	Pump Design
This Work	424.7	7.1	5.6	14	45	2524	356.26	5.94	Capsule (New)
		4.2			45	1531	364.52	3.60	
		2.9			45	195	66.96	0.46	
Benard 1998 [15]	35.28		-	0.53	60–75	50	-	1.42	Diaphragm
Sassa 2011 [13]	27.61	2.8	1.8						
Guo 2008 [18]	40,500	3	8	-	80	1000	333.33	0.02	Peristaltic
Dong 2001 [16]	54	1.1	0.006	-	70–75	340	321.97	6.30	Membrane
Makino 2001 [17]	280		0.095	-	80	4.8	-	0.02	Membrane
Sun 2008 [20]	67,200	1.4	5.8		50	1000	694.44	0.01	Peristaltic

* SHP = Static Head Pressure.
